# Fabrication of antibacterial silver nanoparticle-loaded PVA/chitosan microfibers *via* forcespinning for wastewater disinfection

**DOI:** 10.1039/d6ra01428c

**Published:** 2026-05-01

**Authors:** Vu Viet Linh Nguyen, H. Long Nguyen, Duc-Quang Hoang, Dai Phu Huynh

**Affiliations:** a Faculty of Applied Sciences, Ho Chi Minh City University of Technology and Engineering 1 Vo Van Ngan, Thu Duc Ward Ho Chi Minh City 700 000 Vietnam linhnvv@hcmute.edu.vn; b Faculty of Materials Technology, Ho Chi Minh City University of Technology, VNU-HCM 268 Ly Thuong Kiet, Dien Hong Ward Ho Chi Minh City 700 000 Vietnam; c Vietnam National University Ho Chi Minh City Linh Xuan Ward Ho Chi Minh City 700 000 Vietnam

## Abstract

Water pollution has become a serious environmental problem in many countries of Southeast Asia, affecting both civil and industrial wastewater. Various research approaches have been employed to purify wastewater, such as using absorbent membranes for heavy metal ions and dyes as well as disinfection using antimicrobial agents. This study aims to fabricate antibacterial polyvinyl alcohol/chitosan (PVA/CS) microfibers containing silver nanoparticles (Ag-NPs) using the forcespinning method. The results of our systematic investigation indicate that experimental factors such as PVA/CS concentration, microfiber injection needle size, and spin speed directly determine the morphology of the fabricated microfibers. The PVA/CS fibers were fabricated with uniform diameters and well-separated silver nanoparticles. The average diameter was estimated to be around (1.74 ± 0.13) µm at a PVA concentration of 13 wt% in a 50% acetic acid solution, with an inner needle diameter of 0.81 mm and a rotational speed of 15 000 rpm. The antibacterial performance was evaluated following the Vietnamese standard TCVN 6187-2:1996 (ISO 9308-2:1990), targeting *Escherichia coli* and coliform bacteria in polluted water. We found that the PVA/CS microfibers loaded with Ag-NPs achieved an antibacterial efficiency of 90%. This strongly hints that PVA/CS microfibers loaded with Ag-NPs can potentially be used as water disinfectants. The outcome of this work implies that PVA/CS microfiber membranes containing AgNPs could be a novel filtering material for wastewater disinfection and could be widely used to purify water for improved public health.

## Abstract 2

Antibacterial forcespun Ag-PVA/CS microfibers (∼1.74 µm) achieved up to 95.4% (*E. coli*) and 93.1% (coliform) bacterial removal from real wastewater samples within 60 min, offering a sustainable and high-throughput solution for water purification.

## Graphical abstract

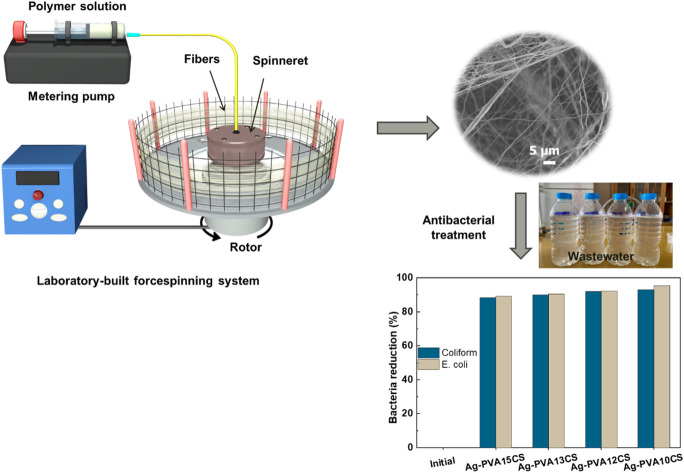

## Introduction

In recent years, silver nanoparticles (Ag-NPs) have attracted a great deal of attention from researchers in various fields, such as biomedicine, electronics, chemical catalysis, and water purification engineering.^1,2^ Ag-NPs have remarkable properties, including physicochemical stability, strong antimicrobial activity, small size and large effective surface area, leading to strong interactions with microorganisms.^3,4^ Such strong interactions are primarily driven by the release of Ag^+^ ions, which disrupt vital cellular processes in bacteria. In addition, Ag-NPs may induce membrane damage and oxidative stress, further enhancing their antibacterial efficacy. Hence, Ag-NPs can be used as disinfectants to eliminate or inhibit the growth of harmful microorganisms, thereby improving the quality of water produced from water treatment processes. Ag-NPs can be embedded in filters or membranes to create a physical barrier that captures or removes bacteria and other contaminants from wastewater and provide long-term antibacterial performance through the release of Ag^+^ ions.^5,6^

Water pollution has been exacerbated by the rapid expansion of industrial activities and urbanization in developing countries, and ineffective management of water resources has a negative impact on our living environment. The contaminants in water can include extremely hazardous chemicals, such as heavy metal ions (Pb^2+^, Cu^2+^, and Cr^6+^), inorganic dyes (methyl/orange blue), or dangerous bacteria such as *Escherichia coli* (*E. coli*) and coliforms. These pollutants actively damage our digestive, circulatory, and excretory systems and can also lead to crucial ecological consequences. Finding an effective and sustainable method to treat the polluted water is therefore necessary and urgent. Various approaches can be used to purify polluted water, such as absorbent membranes for heavy metal ions or inorganic dyes and disinfection with antibacterial agents.^7–9^ A promising method involves combining a biodegradable polymer with Ag-NPs, with the aim of eliminating bacterial contamination. Therein, Ag-NPs are embedded or loaded onto micro-/nano-fibers using techniques such as electrospinning, forcespinning (centrifugal spinning) and melt-blowing.^10–12^

Electrospinning is a simple and low-cost technique, but it still has some limitations for producing materials. This method requires polymer solutions to be compatible with the applied electric field, and solvents to have high volatility and good solubility for polymers.^13,14^ In addition, the production rate of electrospinning is relatively low, which limits its scalability for large-scale applications. In contrast, melt blowing can produce microfibers with a high production rate. However, this method needs high polymer melting temperatures and requires the use of hot air at high speed to produce the micro/nano-fibers.^15,16^ The forcespinning method has recently emerged as an alternative promising approach that can overcome the limitations of the above methods. This can produce large quantities of microfibers from polymer solutions without using heat or high voltages/electric fields. Herein, polymer jets are ejected and elongated into fibers by a centrifugal force, enabling the use of both conductive and non-conductive polymer solutions.^14,17,18^ Moreover, this method is also suitable for biodegradable polymers such as polylactic acid (PLA) and chitosan (CS). Such polymers can be solubilized in water/acetic acid, which is suitable for the key criteria of eco-friendly environment and sustainable processing routes.^11,19^

Many research works have demonstrated that the forcespinning method can potentially be utilized to fabricate polymer fibers embedded with Ag-NPs for water purification.^10,12^ As discussed, the forcespinning protocol offers higher production rates and broader ranges of materials and does not require the use of electric fields, resulting in a more scalable and eco-friendly approach.^20–22^ However, systematic studies focusing on the influence of key processing parameters on fiber morphology and their correlation with antibacterial performance in AgNP-loaded PVA/CS systems remain limited. Based on the said promising features, we aim to fabricate PVA/CS microfibers incorporating Ag-NPs using the forcespinning method, focusing on optimizing the morphological and antibacterial properties of the fabricated PVA/CS microfibers incorporated with Ag-NPs, potentially leading to sustainable water purification applications.

Herein, the Ag-NPs incorporated into PVA/CS microfibers were fabricated using the forcespinning method. The effects of typical process factors, such as concentration of PVA/CS, needle diameter, and spinning speed, on the morphology of the microfibers were systematically investigated. Moreover, the antibacterial performance of the grown fibers was examined following the Vietnamese standard TCVN 6187-2:1996 (ISO 9308-2:1990) by determining the presence and concentration of *E. coli* and coliform bacteria in contaminated water samples. The correlation of the Ag-NPs incorporated in the PVA/CS microfibers and the morphology of those fibers, with respect to the antibacterial activity of the AgNP-loaded PVA/CS fibers, is emphasized. In this study, the incorporation of AgNPs is primarily inferred from the antibacterial performance and morphological observations. Details of the quantitative analysis and elemental mapping are beyond the scope of the current work and will be addressed in future studies. We establish a foundation for optimizing biodegradable polymer-based antimicrobial fibers as a promising platform for sustainable and eco-friendly water-purification applications.

## Experimental

### Materials

PVA with an average molecular weight (*M*_w_) of 85 000–124 000 g mol^−1^, a hydrolytic capacity of 87–89%, and a viscosity of 23–27 cP was supplied by Sigma-Aldrich, Singapore. CS with a viscosity of >1000 cP and a deacetylation of >75% was provided by Vinafood, Vietnam. Acetic acid and ethanol with purities of >98% were purchased from Xilong, China. Ag-NPs with a concentration of 1000 ppm were purchased from AHT Corp., Vietnam. All chemicals were of analytical grade and were used directly without purification.

### Forcespinning PVA/CS and AgNP-loaded PVA/CS microfibers

**Fig. 1:**
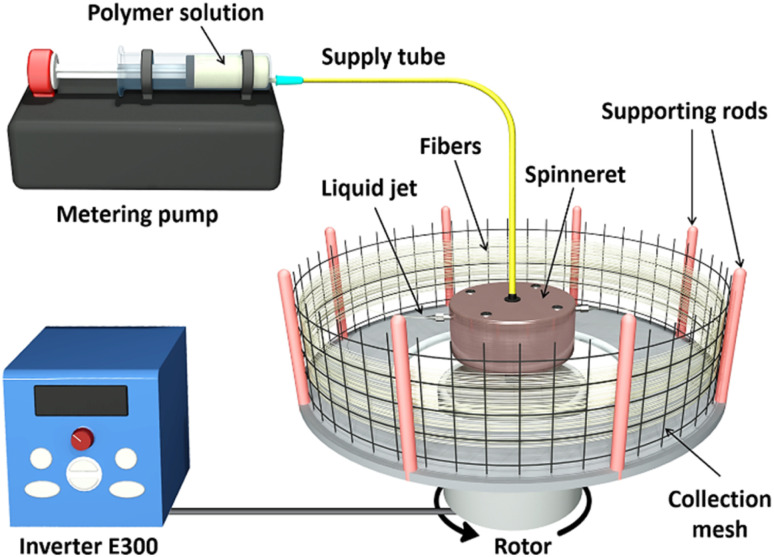
Schematic of the standard process used to fabricate PVA/CS microfibers and Ag-PVA/CS with the laboratory-built forcespinning system.

As the first step of the forcespinning method, a mixture of PVA and CS with a CS/PVA ratio of 5 wt% was mechanically stirred in 50 wt% acetic acid (AA) at ambient temperature for 2.5 h to obtain a homogenous solution. Ethanol was then added to improve solubility, and the solution was continuously stirred for 30 min. The quasi-final PVA/CS solution was stabilized at 25 °C for 3 h to completely remove the air bubbles. The final PVA/CS solution was loaded into the laboratory-built forcespinning system and used to produce spun fibers, as described in Fig. 1. The fabrication system consists of a needle-based spinneret, made from stainless needle tips with different gauges (18G, 20G, and 22G), corresponding to manufacturer-specified inner diameters (ID) of 1.27, 0.81, and 0.64 mm, respectively. The rotor speed was controlled by an E300 inverter through a frequency modulation tool. The inverter provides an adjustable frequency range of 0–400 Hz, corresponding to a rotor speed range of 0–2.4 × 10^4^ rpm. The pumping meter ensures a precise flow rate of 12 mL min^−1^ of the PVA/CS solution into the spinneret. A 10 cm distance from the needle tip to the collectors, including supporting rods and collection meshes, was fixed for all experiments. During the centrifugal spinning, the solution was moved from the needle to the collector, and the solvent evaporated on the collector as solid fibres. The forcespinning process was carried out at ambient temperature, and the resultant PVA/CS fibres were then dried at room temperature for 24 h to completely eliminate solvent.

The Ag-NP-loaded PVA/CS (Ag-PVA/CS) solutions were prepared as described for the pristine PVA/CS solutions. Therein, PVA, CS, and AA were mixed to produce polymer forcespinning solutions with different PVA concentrations (10–15 wt%) in total polymer solution, while maintaining a constant CS/PVA ratio of 5 wt%. A total of 100 g of the polymer mixture containing PVA + CS + AA + ethanol was used for each formulation, and 10 g of an Ag-NPs aqueous dispersion with a fixed concentration of 100 ppm was incorporated into each formulation, aiming to impart antibacterial functionality. The compositions of each formulation are detailed in Table 1. The resultant Ag-PVA/CS solutions were subsequently used in our laboratory-built forcespinning system to fabricate microfibers, as described in Fig. 1.

**Table 1:** Compositions of the precursor PVA/CS solutions with and without Ag-NPs (100 ppm) used for spinning polymer microfibers in our forcespinning system^a^

Sample codes	PVA (g)	CS (g)	AA (g)	Ethanol (g)	Ag-NPs (100 ppm) (g)
PVA10CS	10	0.50	84.50	5	0
Ag-PVA10CS	10	0.50	84.50	5	10
Ag-PVA11CS	11	0.55	83.45	5	10
Ag-PVA12CS	12	0.60	82.40	5	10
Ag-PVA13CS	13	0.65	81.35	5	10
Ag-PVA15CS	15	0.75	79.25	5	10

a The sample codes denote the PVA wt% in the total polymer solution, while the Ag-NP concentration was fixed at 100 ppm for all Ag-PVA/CS samples.

### Characterization

The morphological characteristics of PVA/CS and Ag-PVA/CS microfiber samples were examined by field-emission gun scanning electron microscopy (FEG-SEM) with a Hitachi FEG-SEM S-4800 (Japan). The electron beam of the FEG-SEM S-4800 was extracted and accelerated at a voltage of 10 keV. From the recorded SEM images, the average diameter of the spun fibres was estimated using ImageJ software.^23^ The average diameters of the spun fibres were also analyzed using the Minitab Statistical Software to determine statistically the mean/error distribution of the microfibers.^24^ The correlations of such data, *e.g.*, frequency and diameter, were plotted using OriginPro.^25^ The viscosity of the PVA/CS solutions with and without Ag-NPs in AA solutions was measured using a Brookfield DV-II+ Pro Viscometer (USA). Such measurements were carried out at a controlled temperature of 25 °C. Each 40 mL solution sample was contained in a standard cylindrical beaker, and all samples reached equilibrium before testing. The viscometer was operated at a rotational speed of 60 rpm for all measurements.

### Antibacterial effects of the spun Ag-PVA/CS fibres

**Fig. 2:**
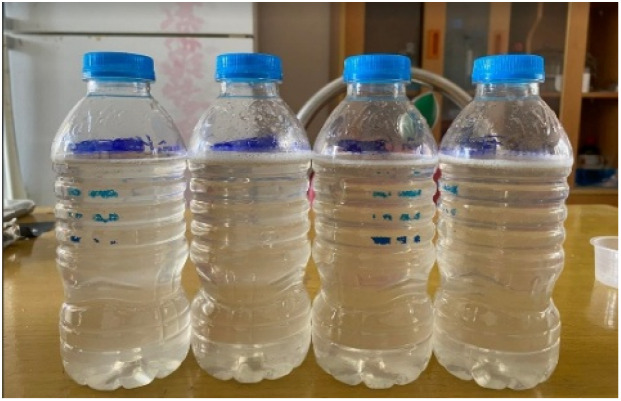
Wastewater samples were collected from the Lo-Gom Canal in Ho Chi Minh City, Vietnam, and stored in plastic containers.

Wastewater samples were collected from the Tan Hoa-Lo Gom Canal in Ho Chi Minh City, Vietnam. The collected wastewater samples were stored in plastic containers, as shown in Fig. 2. Sampling, preservation, and handling procedures were carefully conducted prior to microbiological analysis. The pH level of the wastewater samples was adjusted to 6.0 using a 5% AA solution.

The concentration of total coliforms and *E. coli* was determined using the Most Probable Number (MPN) method specified in TCVN 6187-2:1996 (equivalent to ISO 9308-2:1990).^26^ These analyses were performed by the Vietnam Environmental Monitoring and Analysis Center (VIMCERTS 197, Vietnam). Briefly, aliquots of undiluted and diluted samples were inoculated in the lactose medium contained in Durham tubes and incubated at 35–37 °C for 24–48 h. Bacteria in the samples metabolize lactose and produce acid and gas. The formation of those acids and gases indicated that bacteria grew positively. Those positive tubes were then compared with the standard MPN tables to statistically estimate the density of bacteria, the so-called MPN/100 mL.

The antibacterial activity of the Ag-PVA/CS fibers against *E. coli* and coliform bacteria was evaluated by immersing 100 mg of spun fibers into 100 mL of wastewater for each sample for 60 min. In this study, antibacterial performance was evaluated at a fixed contact time of 60 min, according to the standard method. Time-dependent antibacterial kinetics and statistical analysis were not investigated and will be addressed in future work. The bacterial concentration before and after treatment was determined using TCVN 6187-2:1996, and the antibacterial efficiency, *R* (%), was estimated using the equation^27^ described below.1
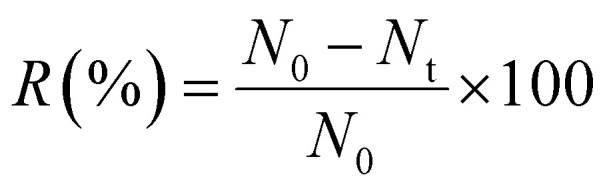
where *N*_0_ and *N*_t_ are the bacterial concentrations (MPN/100 mL) before and after treatment, respectively.

## Results and discussion

### PVA concentrations affecting spun fiber structures and PVA/CS solution viscosity

Polymer concentration significantly impacts bead formation, fiber alignment, and the diameter of the fabricated fibers. Therein, beads are usually formed at a low concentration, while the intermixing of bead and fiber geometries could occur at higher concentrations. Continuous fibers are developed when sufficient polymer chain entanglement occurs at critical polymer concentrations.^28^ Optimal PVA concentration can suppress the formation of beads on the fibers, leading to the generation of fibers without any beads attached to them. Hence, beaded fibers can be formed at concentrations above the optimal one. By increasing the PVA concentration and/or the molecular weight in the polymer solution, the degree of polymer chain entanglement in the polymer solution is enhanced, resulting in larger fiber diameters.^29^ However, some typical processing factors of our forcespinning system were fixed, *i.e.*, a flow rate of 12 mL min^−1^, the distance between the spinneret and collector of 10 cm and a rotation speed of 15 000 rpm, for the given study.

**Fig. 3:**
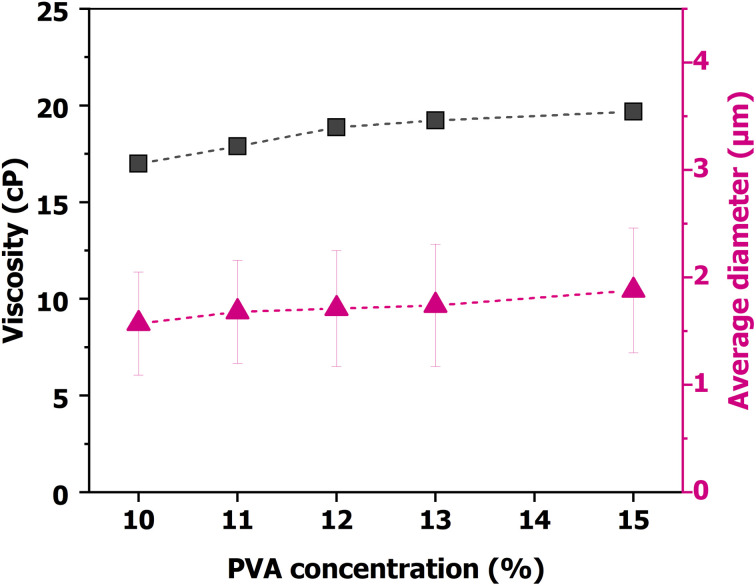
Viscosity of the PVA/CS solution (cP) and the average diameters of the microfibers (µm) as a function of PVA/CS concentrations (wt%) that were used in our laboratory-built forcespinning system.

**Fig. 4:**
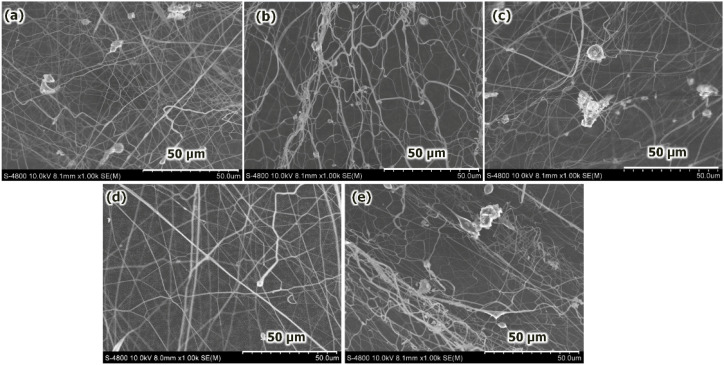
FEG-SEM images of the PVA/CS force-spun fiber surfaces fabricated using differing PVA concentrations: (a) 10 wt%; (b) 11 wt%; (c) 12 wt%; (d) 13 wt%; and (e) 15 wt%. The fabrication conditions were a rotational speed of 15 000 rpm; a needle with an ID of 1.27 mm; and a collection distance of 10 cm.

As stated, the compositions of the PVA/CS precursor solutions with and without Ag-NPs used for producing the polymer microfibers are listed in Table 1. The viscosity and morphology of those samples were characterized, as presented in Fig. 3 and 4, respectively, and also detailed in Table 2. The obtained data show that the viscosity of the PVA/CS solution increases steadily from 16.99 cP (10 wt%) to 19.68 cP (15 wt%). The viscosity measurements were performed once for each sample, and thus no standard deviation is reported. The viscosity values are presented to evaluate the influence of solution properties on fiber formation during the forcespinning process. A consistent increase in viscosity with PVA concentration is observed, which correlates well with the transition in fiber morphology from bead-containing structures to smooth fibers and then to irregular structures. The viscosity increased, leading to enhanced polymer chain entanglement and stronger hydrogen-bond interactions between the PVA and chitosan molecules in the acetic acid medium. Such rheological changes directly influence the morphology of fibers during the forcespinning process. However, at a low viscosity level of ∼17 cP (10 wt%), where the bonding interaction of molecules is weak, unstable jet formation occurs, leading to beads and disc-like structures, as shown in Fig. 4(a). Increasing the viscosity value to ∼19 cP (12–13 wt%) leads to the formation of a stable polymer jet, yielding uniform and continuous fibers with minimal bead defects, as shown in Fig. 4(b–d). For fiber diameter measurements, each condition was evaluated over multiple fibers, and the error bars shown in Fig. 3 reflect the standard deviation of these measurements. This behavior suggests that the effect of polymer concentration prevailed over minor variations in viscosity, and the observed trend is sufficient to explain the change in fiber morphology.

**Table 2:** Viscosity values of the PVA/CS solutions with different concentrations and the morphological characteristics of force-spun fibers characterized by SEM

PVA (wt%)	Viscosity (cP)	Average fiber diameter (µm)	Fiber morphology (SEM)
10	16.99	1.57 ± 0.48	Beads + discs
11	17.89	1.68 ± 0.48	Fewer beads
12	18.87	1.71 ± 0.54	Smooth fibers
13	19.23	1.74 ± 0.57	Smooth fibers
15	19.68	1.88 ± 0.58	Irregular, nonuniform

Upon further increasing the viscosity values to >19.5 cP (15 wt%), the stretchability of the jet is reduced. This leads to an intermittent clogging of the nozzle during the forcespinning process, resulting in nonuniform fibers, and the appearance of beads during fiber formation is also noted, as revealed in Fig. 4(e). The viscosity of PVA/CS solutions increased with increasing PVA concentration, as listed in Table 2. Higher viscosity levels, *i.e.*, 18–19 cP, facilitate the formation of a stable polymer jet during the forcespinning process. This results in a smoother surface of the fabricated fibers, as shown in the SEM images in Fig. 4. However, the results also indicate that when the viscosity is either too high or too low, unstable polymer jets can form, leading to heterogeneous morphology of the resultant fibers. The trend described for the given behavior is seen in Fig. 3, where the average fiber diameter and viscosity increase quasi-linearly with increasing PVA/CS concentration. This indicates that the rheological properties of the PVA/CS solution strongly govern the morphology of fabricated fibers during the forcespinning process. Moreover, viscosity increases with increasing PVA concentration, leading to stronger linking of fiber chains, producing thicker and more uniform fibers. The optimal 13 wt% concentration provides a balanced viscoelasticity, allowing continuous fiber formation without the appearance of granular/bead defects during the forcespinning process.

As mentioned above, SEM images indicate the presence of many beads/discs along with the microfibers on the sample surface fabricated at a PVA concentration of 10 wt%, as seen in Fig. 4(a). When the PVA/CS concentration increased up to the range of 11–12 wt%, the number of beads/discs reduced significantly, as clearly observed in Fig. 4(b and c). Particularly, continuous and homogeneous microfibers were obtained at a PVA/CS concentration of 13 wt% (Fig. 4(d)). This suggests that 13 wt% is a critical value for the PVA/CS concentration that allows sufficient polymer chain entanglement in the jet during the forcespinning process. Upon increasing the polymer concentration to 15 wt%, bead/disc droplets appear again on the force-spun microfiber surfaces. This could be understood by considering that the surface tension of the polymer solution is too high at this concentration, leading to the ratio of surface area to mass being decreased. This increases the resistance to the polymer jet flow toward the collector of the forcespinning system. Furthermore, the insufficient surface-to-volume inhibits solvent evaporation, thereby leading to the formation of droplets instead of continuous microfibers. Based on these observations, we found that a PVA/CS concentration of 13 wt% is optimal for our laboratory-built forcespinning system; with concentrations lower or higher than the optimal conditions, the needle becomes clogged due to lower or higher viscosity, making the formation of smooth microfibers difficult. Further studies on the dynamics of beads or disc formation at different concentrations. particularly in the relation to the self-cleaning behavior of geometrical needles to prevent clogging, have been reported by several research groups;^30^ however, this is beyond the scope of the current work.

### The effects of processing parameters on the force-spun fiber morphology

The force spun fibers fabricated from low-concentration polymer solution are short and tend to adhere to each other, forming overlapped and entangled structures. As the PVA concentration increases, the fibers become more separated and elongated. Hence, if the rotation speed of the spinneret increases, the concentration should be simultaneously increased, allowing the spun fibers to be spread evenly across the surface as uniform microfibers.

**Fig. 5:**
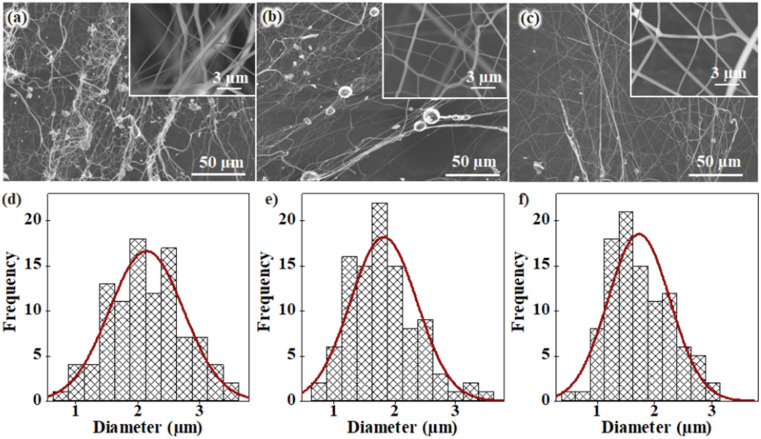
(a–c) Typical FEG-SEM images and (d–f) statistical size distribution plots of the force-spun microfibers, fabricated at a PVA/CS concentration of 13 wt%, using a needle with an ID of 1.27 mm and a collection distance of 10 cm. The centrifugal speeds were varied at (a and d) 9000 rpm, (b and e) 12 000 rpm, and (c and f) 15 000 rpm.

As a key experimental factor, the spinneret rotation speed directly determines the magnitude of the centrifugal force and affects the forming capacity, configuration, and diameter of the force-spun fibers. Hence, the effects of spinneret rotation speeds on the morphology and diameter of force-spun fibers fabricated by our laboratory-built forcespinning system were investigated. Herein, the centrifugal speeds were varied to 9000 rpm, 12 000 rpm, and 15 000 rpm, and FEG-SEM images of the fabricated samples were recorded, as presented in Fig. 5(a–c), respectively. As clearly seen, beaded fibers were observed with lower rotation speeds of 9000 rpm or 12 000 rpm (Fig. 5(a and b)), while continuous fibers with a homogeneous surface were obtained at a higher rotation speed of 15 000 rpm. Based on the SEM images recorded at a low magnification, the diameter distributions of the force-spun microfibers were statistically calculated from 100 particles per fibers, as shown in Fig. 5(d–f). The mean and standard deviation values of those 100 particles per fibers were calculated. As a result, the average diameters of the force-spun fibers were obtained as (2.00 ± 0.66) µm, (1.81 ± 0.55) µm, and (1.72 ± 0.54) µm at rotation speeds of 9000 rpm, 12 000 rpm, and 15 000 rpm, respectively. Such values clearly show that the fiber diameter gradually reduces as the centrifugal speed of the needle increases.

The above results can be interpreted as a mechanism in which the liquid-to-solid transition during the forcespinning process can be separated into two simultaneous processes: (i) solvent diffuses from the center to the surface of the fiber, and (ii) solvent evaporates on the fiber surface. Hence, when the rotation speed of the spinneret is reduced, its centrifugal force reduces significantly, causing the stretching force on the fibers to decrease, resulting in fibers that are unstretched and exhibit large diameters.^13,30^ As a result, the diameter of the fibers increases, the diffusion time of solvent from the center to the surface of a fiber increases, and the solvent is unable to evaporate effectively. In addition to the stretching effect of the polymer solution, the centrifugal force also affects the molecular chains, preventing clustering and rearranging of the molecular chains to reduce surface tension. Therefore, as the rotation speed of the spinneret is increased, it reaches a sufficiently high centrifugal force to prevent the clustering formation of molecular chains, which will rearrange the molecular chains and reduce surface tension. Subsequently, the spun microfibers will be straight, and the formation of ‘bead fibers’ is restricted. Hence, smooth fibers with smaller diameters and a homogeneous morphology are fabricated at a rotation speed of 15 000 rpm. The centrifugal force is strong enough to stretch/orient the polymer flow to form smooth fibers. Moreover, the polymer molecular chains are significantly affected by the centrifugal force, suppressing their ability to move or rearranging them during the forcespinning process. Based on the morphology and diameter characteristics of the spun microfibers, the optimal rotation speed of the spinneret is found to be 15 000 rpm; this condition was used for the subsequent investigations.

**Fig. 6:**
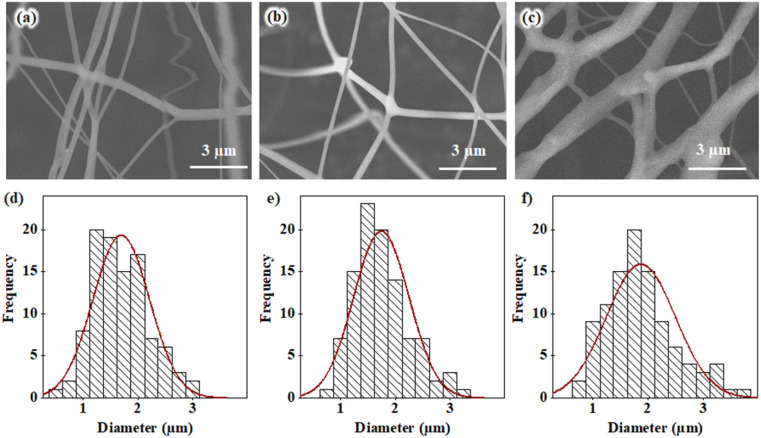
(a–c) FEG-SEM images of PVA/CS force-spun surfaces, and (d–f) fiber diameter distribution histograms of three typical samples fabricated with a PVA/CS concentration of 13 wt%, a rotational speed of 15 000 rpm, a collection distance of 10 cm, and needles with different inner diameters of (a and d) 0.64 mm; (b and e) 0.81 mm; and (c and f) 1.27 mm.

Another physical factor affecting the forcespinning process of the polymer solution flows in our fabrication system is the diameter of the needles. Herein, the diameter strongly induces the initial solution stream flowing in the needle, leading to changes in the diameters of microfibers. Three different needle diameters, 0.64 mm, 0.81 mm and 1.27 mm, were used, and SEM images of the generated fiber surfaces were recorded. Three typical FEG-SEM images of the spun fiber samples are shown in Fig. 6(a–c), and the statistical diameter distributions of those spun microfibers were also analyzed, as given in Fig. 6(d–f). The results show that the average diameters of the spun microfibers increase slightly from (1.70 ± 0.52) µm to (1.87 ± 0.63) µm when the needle diameter increases from 0.64 mm to 1.27 mm. Hence, when a larger needle is used, the initial volume of the polymer solution flowing out of the tip is larger with the same centrifugal force. Based on the morphology and diameters of the spun fibers, the uniform fibers with respect to shape and geometry were fabricated when the 0.81 mm diameter needle was used; this needle was therefore used for further experiments.

In fact, the interaction of intermolecular chains induces significant effects with respect to stretching in the polymer flow under the centrifugal force. As a result, the spun fibers were created with larger diameters. For the PVA/CS solution with high viscosity, the viscosity changes more significantly than in lower-viscosity solutions. Hence, choosing an appropriate centrifugal force/speed is a crucial task because the centrifugal force stretches the polymer solution during the forcespinning process. Herein, a centrifugal speed of 15 000 rpm was used for our fabrications.

### Effects of PVA concentrations on the surface and diameter of the Ag-PVA/CS fibers

**Fig. 7:**
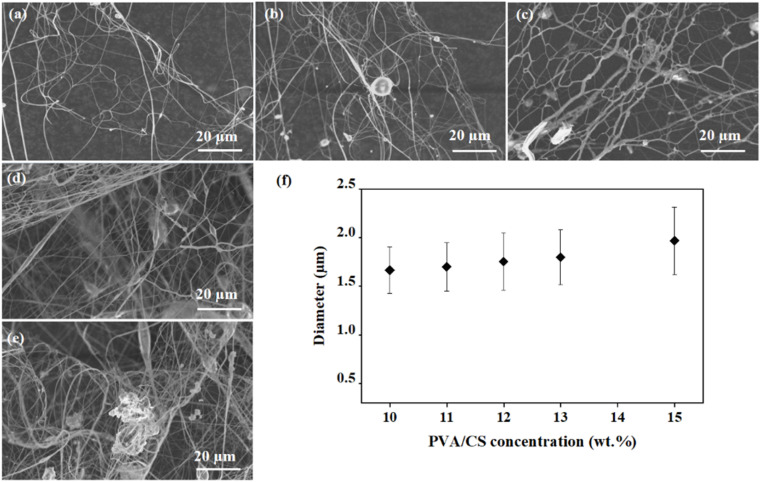
SEM images of the force-spun Ag-PVA/CS fiber sample surfaces. The samples were fabricated at a rotational speed of 15 000 rpm, a needle diameter of 1.27 mm, a collection distance of 10 cm, and a flow rate of 12 mL min^−1^, with differing PVA/CS concentrations of (a) 10 wt%, (b) 11 wt%, (c) 12 wt%, (d) 13 wt% and (e) 15%. (f) The average diameters of 100 fibers were calculated and plotted as a function of the PVA/CS concentrations.

Following the same fabrication and data analysis protocols described in “The effects of processing parameters on the force-spun fiber morphology”, SEM images of the Ag-PVA/CS spun fibers fabricated at different PVA concentrations, increasing from 10 wt% to 15 wt%, are given in Fig. 7(a–e). The average fiber diameters were calculated and are plotted in Fig. 7(f). The results show that the average fiber diameter gradually increases with increasing solution concentration from 10% to 15%, ranging from (1.62 ± 0.48) µm to (1.97 ± 0.69) µm. Among those experimental data points, an average fiber diameter of (1.62 ± 0.48) µm was achieved at the lowest PVA/CS concentration of 10 wt%; when the PVA/CS concentrations were increased to 11, 12, and 13 wt%, their diameters increased to (1.70 ± 0.50) µm, (1.75 ± 0.57) µm, and (1.79 ± 0.58) µm, respectively. An average fiber diameter of (1.97 ± 0.69) µm was reached at 15 wt% PVA/CS concentration. Such results indicate that higher PVA/CS concentrations led to thicker spun fibers. This outcome is consistent with the fact that the viscosity of the spinning solutions is enhanced when the rotational speed is increased.

As seen in Fig. 7(f), the average fiber diameters gradually expand when the PVA/CS concentration is increased, which could be attributed to the increase in viscosity and/or enhanced intermolecular interactions within the spinning solution. In fact, the density of polymer chain entanglement increases with increasing the PVA concentration, which subsequently increases the resistance to stretching duringthe forcespinning process. Consequently, the solution jets undergo less elongation and create thicker fibers. Moreover, interactions within the polymer chains are stronger in polymer solutions with higher concentrations, which restricts the stretching of the polymer jets under centrifugal force. This suggests that the polymer jets exhibit limited elongation, leading to the formation of fibers with large diameters. As the polymer viscosity increases, the solution displays greater resistance to deformation, which attenuates the elongational effect of centrifugal force on the jet.

### Antibacterial properties of the spun Ag-PVA/CS microfibers

To examine the antibacterial performance of the Ag-PVA/CS spun fibers with different PVA weight percentage (wt%) against *E. coli* and coliform bacteria, 100 mg of the fabricated samples were immersed in 100 mL of the collected wastewater for 60 min. The bacterial concentrations (MPN/100 mL) before and after treatment were then calculated; the results are presented in Table 3. When the PVA content was decreased from 15 wt% to 10 wt%, the antibacterial performance of the Ag-PVA/CS spun fiber samples toward both *E. coli* and coliform bacteria increased slightly. In particular, the antibacterial performance is superior at 10 wt% PVA concentration, reaching 93.1% and 95.4% for coliform and *E. coli*, respectively.

**Table 3:** Antibacterial performance of the Ag-PVA/CS spun fibers with different PVA wt% against *E. coli* and coliform bacteria present in the samples collected from wastewater sources

Samples	Coliform (MPN/100 mL)	*E. coli* (MPN/100 mL)	Coliform reduction (%)	*E. coli* reduction (%)
Initial wastewater	93.0 × 10^6^	24.0 × 10^5^	—	—
PVA/CS	—	—	—	—
Ag-PVA15CS	11.0 × 10^6^	2.6 × 10^5^	88.2	89.2
Ag-PVA13CS	9.3 × 10^6^	2.3 × 10^5^	90.0	90.4
Ag-PVA12CS	7.5 × 10^6^	1.9 × 10^5^	91.9	92.1
Ag-PVA10CS	6.4 × 10^6^	1.1 × 10^5^	93.1	95.4

**Fig. 8:**
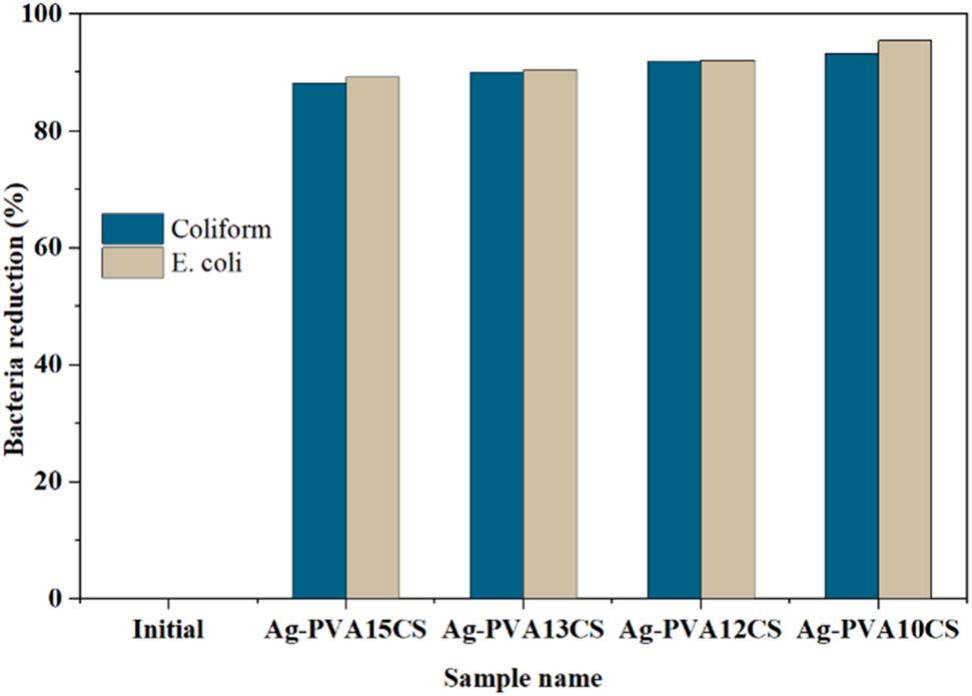
Correlation of the antibacterial performance or bacterial reduction (%) of the Ag-PVA/CS spun fibers with different PVA wt% against coliform and *E. coli* in the collected wastewater samples.

The antibacterial performance of the spun-fiber samples with different Ag-NP concentrations targeting coliform and *E. coli* in our collected wastewater samples is plotted in Fig. 8. All samples exhibited strong activity against both coliform and *E. coli* bacteria, with high bacterial reduction values in the range of 88.2–95.4%. Based on the data obtained from the four wastewater samples, we can conclude that the antibacterial efficiency increases with decreasing fiber diameter, which enhances the effective surface area and promotes greater interaction between the fibers and the bacteria. While the initial AgNP content was kept constant, the variation in fiber morphology appears to play a more dominant role in determining antibacterial performance. Because the Ag-PVA/CS spun fiber fabricated with 10 wt% PVA concentration presents a smaller fiber diameter, as seen in Fig. 7(f), it has a larger effective area, as compared to the sample fabricated with 15 wt% PVA. Due to the increase in the effective surface area, the Ag-PVA/CS spun fibers have greater contact with the bacteria, leading to increased antibacterial efficiency.

A comparison of AgNP-loaded fibers and chitosan-based fibrous systems, including fiber diameter, Ag loading, antibacterial performance, and contact time, is presented in Table 4. The results show that the Ag-PVA/CS fibers exhibit competitive antibacterial performance while employing a scalable forcespinning production technique.

**Table 4:** Qualitative comparison of Ag-loaded fibrous systems in terms of fiber diameter, antibacterial performance, and contact time of samples^a^

Material system	Fabrication method	Fiber diameter	Ag loading in fiber	Antibacterial performance	Contact time of samples	Ref.
Polyvinylidene fluoride (PVDF)/polyvinyl pyrrolidone (PVP)/AgNPs	Electrospinning	∼500 nm	Not reported	Inhibition zone against *S. aureus* (not reported antibacterial reduction)	Not report	10
PLA/chitosan-based silver nanoparticles (AgCH-NPs)	Forcespinning	5–20 µm	Not reported	Inhibition efficiency against *S. aureus* 90%	24 h	11
PEO/AgNPs	Centrifugal spinning	∼232 nm	Not quantified (confirmed by EDS and TGA)	Inhibition efficiency against *E. coli* (99%) and *B. cereus* (88%)	24–168 h	12
PVA/carboxymethyl CS fibers	Centrifugal spinning	1.5–3.3 µm	Not reported	Inhibition efficiency against *S. aureus* (72%) and *E. coli* (21%)	7 h	29
PVA/CS/AgNPs	Forcespinning	1.6–2 µm	Not quantified	Antibacterial reduction of *E. coli* (95%) and coliform (93%)	60 min	This work

a Ag loading was not quantified in this work due to the absence of ICP-OES or TGA.

It can be observed that most literature reports evaluate antibacterial efficiency using inhibition zones or metabolic assays, which provide indirect measurements. In contrast, the present PVA/CS/AgNP fibers were assessed using the Most Probable Number (MPN) method, enabling a direct and quantitative estimation of bacterial reduction. The PVA/CS/AgNP fibers developed in this work show 88.2–95.4% antibacterial reduction of *E. coli* and coliform within 60 min, which is comparable to or better than previously reported systems with similar fiber diameters. The incorporation of chitosan not only contributes intrinsic antibacterial properties but also facilitates a synergistic effect with AgNPs, enhancing the overall efficacy. Furthermore, the use of a scalable forcespinning technique allows practical production advantages over conventional electrospinning.

The antibacterial mechanism of CS mainly comes from the interactions of polycations in CS and the negatively charged surfaces of the bacteria. Therein, the protonated amine groups of CS actively interact with anions such as lipopolysaccharides and proteins at the Gram-negative cell surfaces.^31,32^ Such interactions may lead to the formation of bonds between the chitosan and the cell membranes, leading to compromised membrane permeability or leakage of intracellular contents, resulting in the destruction of bacterial cells. This suggests that pristine PVA/CS fibers may exhibit a certain level of antibacterial activity due to the presence of chitosan, although lower than AgNP-loaded systems.^31,33^ The antibacterial activity of AgNP-loaded fibers is generally attributed to the release of Ag^+^ ions into the aqueous environment, which interact with bacterial cells and disrupt their metabolic processes. In polymeric systems such as PVA/CS, the matrix may regulate the diffusion of Ag^+^ ions, potentially contributing to a sustained antibacterial effect.

The said process can also be fundamentally understood through another mechanism, wherein the negatively charged phospholipid bilayers on the bacterial cell membranes play a crucial role.^34,35^ If exposed to a surrounding environment containing positively charged Ag-NPs (Ag^+^), the permeability of the cell membrane is altered, causing a change in its surface charge, such as charge reversal or membrane inactivation. As a result, the protein layers in the cell membrane are denatured, or the phospholipid bilayer is directly exposed. This is generally considered to be one of the main factors contributing to cell membrane instability, where the membrane is broken, leading to leakage of intracellular substances and ultimately bacterial cell death.^36^ This antibacterial mechanism is mainly attributed to the electrostatic attraction between positively charged Ag^+^ and negatively charged bacterial membranes, followed by interaction with intracellular thiol-containing enzymes, which disrupts membrane integrity, cellular respiration, and essential metabolic pathways. At this stage, the Ag^+^ ions are adsorbed onto the bacterial surfaces and penetrate into the bacterial cells, finally neutralizing or completely eliminating the bacteria.^37^ For our Ag-PVA/CS spun-fiber samples, Ag^+^ ions will kill bacteria by attacking the cell membrane at the thiol (–SH) groups of the membrane proteins and respiratory enzymes, preventing the oxygen transport process from outside into the cell, ultimately destroying the bacteria.^38^ Due to the strong affinity of Ag^+^ to sulfur-containing groups, stable Ag–S bonds are formed, leading to enzyme inactivation and inhibition of the respiratory chain. Such a process reduces ATP production and also prevents gene replication.^39^ However, the release profile of Ag^+^ ions as a function of time was not investigated in this study and should be addressed in future work to better understand the long-term antibacterial behavior and environmental impact. It should be noted that the proposed antibacterial mechanisms are based on literature reports, and no direct ROS or membrane imaging measurements were studied in this work. Furthermore, the structural integrity and antibacterial effectiveness of the fibers after multiple cycles were not investigated. Future studies will focus on evaluating fiber stability and reusability to better understand their practical and economic feasibility.

## Conclusions

We successfully fabricated antibacterial Ag-PVA/CS microfibers using our laboratory-built forced-spin system. Three physical parameters used in the material fabrication process were systematically investigated for the given fabrication system. These are the PVA/CS concentration, solution injector diameter, and rotational speed, which directly determine the structural morphology of the fabricated microfibers. We found that a PVA/CS concentration, solution injector diameter, and rotational speed of 13 wt%, 1.27 mm, and 1.5 × 10^4^ rpm, respectively, were optimal. Using these optimized values, the structural properties of the fabricated microfibers are suitable for many potential applications. In particular, the PVA/CS microfibers containing Ag-NPs fabricated under the optimal conditions exhibited an average fiber diameter of around 1.74 µm. Moreover, our microfiber samples all exhibited good antibactericidal activity, achieving antibactericidal efficiency of 88.2–95.4% for both coliform and *E. coli* bacteria in contaminated water samples collected in Vietnam. Such preliminary data discussed in this work provide a promising foundation for further research to develop novel disinfectant materials with a high antibactericidal efficiency, potentially eliminating harmful bacteria in contaminated water. Our Ag-PVA/CS microfibers could be considered a potential filtration material that could be used to filter and decontaminate polluted water in Vietnam and improve the public health of our community. However, further studies on Ag^+^ release behavior, time-dependent antibacterial testing, and flow-through experiments are required to fully evaluate its practical applicability. In addition, a comparative study with free AgNPs in solution was not conducted in this work and should be considered in future studies to further elucidate potential synergistic effects.

## Author contributions

Conceptualization: D. P. Huynh; supervision: D. P. Huynh; methodology: D. P. Huynh, H. L. Nguyen; investigation: H. L. Nguyen; data curation: V. V. L. Nguyen; formal analysis: V. V. L. Nguyen; visualization: V. V. L. Nguyen, D. Q. Hoang; writing-original draft: V. V. L. Nguyen; and writing-reviewing and editing: D. Q. Hoang, V. V. L. Nguyen.

## Conflicts of interest

The authors declare that they have no conflict of interest.

## Data Availability

All data supporting this study are included in this article. Additional data are available from the corresponding author upon reasonable request.
